# Supplementary material for: Peripheral blood inflammatory cytokines linked to clinical outcomes in Mycoplasma pneumoniae pneumonia
Source: Microbiol Spectr. 2025 Nov 28;14(1):e01615-25. doi: 10.1128/spectrum.01615-25 (PMC12772330; doi:10.1128/spectrum.01615-25)
Supplement: Supplemental material — Fig. S1 and S2; Tables S1 and S2. [file spectrum.01615-25-s0001.docx]

### Supplementary Materials

Tab S1: Admission characteristics of children with Mycoplasma pneumoniae pneumonia in the retrospective cohort according to their subsequent clinical outcome.

**A：**Clinical characteristics at admission between necrotizing pneumonia and non-necrotizing pneumonia

| Characteristic | Total MPP(N=2391) | NP（n=51） | Non-NP（n=2304） | P-value |
| --- | --- | --- | --- | --- |
| Age (years) | 7.03 (4.94, 8.67) | 7.40 (6.31, 8.49) | 7.01 (4.93, 8.67) | 0.231 |
| Sex n (%)  Male  Female | 1186 (49.60)  1205 (50.40) | 21 (41.18)  30 (58.82) | 1165 (49.79)  1175 (50.21) | 0.224 |
| Fever, n (%) | 2275 (95.15) | 50 (98.04) | 2225 (95.09) | 0.521 |
| Preadmission fever duration (d) | 7.00 (5.00, 10.00) | 10.00 (7.00, 14.00) | 7.00 (5.00, 10.00) | <.001 |
| CRP, mg/L | 7.19 (2.61, 20.45) | 13.84 (3.60, 40.17) | 7.19 (2.61, 20.45) | 0.054 |
| WBC, ×10^9^/L | 9.66 (7.17, 12.86) | 12.25 (9.04, 15.07) | 9.62 (7.14, 12.80) | <.001 |
| Hemoglobin, g/L | 126.00 (119.00, 132.00) | 125.00 (116.00, 130.50) | 126.00 (119.00, 132.00) | 0.406 |
| Platelets, ×10^9^/L | 319.00 (236.00, 419.00) | 313.00 (237.00, 423.50) | 319.50 (236.00, 419.00) | 0.921 |
| NLR, % | 246.69(157.25,423.89) | 402.67(211.19,1004.6) | 244.61(157.04,416.33) | <.001 |
| LDH, U/L | 318.00 (270.00, 388.00) | 414.50 (333.25, 536.00) | 316.00 (269.00, 386.00) | <.001 |
| PT, s | 12.00 (11.40, 12.90) | 12.00 (11.20, 13.10) | 12.00 (11.40, 12.90) | 0.804 |
| APTT, s | 31.10 (28.20, 34.30) | 28.00 (25.60, 31.30) | 31.20 (28.30, 34.30) | <.001 |
| D-dimer, ng/mL | 226.00 (149.00, 400.00) | 1994.00 (553.00, 2651.00) | 224.00 (147.00, 387.25) | <.001 |
| Fibrinogen, g/L | 3.34 (2.84, 3.80) | 3.45 (2.56, 4.27) | 3.34 (2.85, 3.80) | 0.396 |
| Pleural effusion, n (%) | 183 (7.65) | 15 (29.41) | 168 (7.18) | <.001 |

**B：**Clinical characteristics at admission between pulmonary embolism and non-pulmonary embolism

| Characteristic | Total MPP（n=2391） | PE（n=20） | Non-PE（n=2371） | P-value |
| --- | --- | --- | --- | --- |
| Age (years) | 7.03 (4.94, 8.67) | 7.19 (6.57, 8.68) | 7.02 (4.93, 8.67) | 0.220 |
| Sex n (%)  Male  Female | 1186 (49.60)  1205 (50.40) | 14 (70.00)  6 (30.00) | 1172 (49.43)  1199 (50.57) | 0.067 |
| Fever, n (%) | 2275 (95.15) | 20 (100.00) | 2255 (95.11) | 0.621 |
| Preadmission fever duration(d) | 7.00 (5.00, 10.00) | 11.00 (7.75, 16.25) | 7.00 (5.00, 10.00) | <.001 |
| CRP, mg/L | 7.19 (2.61, 20.45) | 15.41 (8.52, 46.13) | 7.14 (2.60, 20.29) | 0.011 |
| WBC, ×109/L | 9.66 (7.17, 12.86) | 11.77 (8.59, 13.74) | 9.65 (7.16, 12.84) | 0.138 |
| Hemoglobin, g/L | 126.00 (119.00, 132.00) | 124.00 (115.75, 127.25) | 126.00 (119.00, 132.00) | 0.136 |
| Platelets, ×109/L | 319.00 (236.00, 419.00) | 299.00 (222.75, 371.25) | 320.00 (236.00, 419.00) | 0.335 |
| NLR, % | 246.69(157.25,423.89) | 467.45(267.66,964.20) | 245.53(157.08,419.89) | <.001 |
| LDH, U/L | 318.00 (270.00, 388.00) | 481.50 (425.25, 584.75) | 317.00 (269.00, 386.00) | <.001 |
| PT, s | 12.00 (11.40, 12.90) | 12.40 (11.62, 13.00) | 12.00 (11.40, 12.90) | 0.469 |
| APTT, s | 31.10 (28.20, 34.30) | 28.05 (26.70, 32.17) | 31.10 (28.20, 34.30) | 0.027 |
| D-dimer, ng/mL | 226.00 (149.00, 400.00) | 2038.50 (712.25, 2844.50) | 225.00 (148.00, 395.50) | <.001 |
| Fibrinogen, g/L | 3.34 (2.84, 3.80) | 3.60 (2.84, 4.42) | 3.34 (2.84, 3.80) | 0.212 |
| Pleural effusion, n (%) | 183 (7.65) | 9 (45.00) | 174 (7.34) | <.001 |

CRP C-reactive protein, WBC white blood cells, LDH lactate dehydrogenase, RMPP refractory M. pneumoniae pneumonia, SMPP severe M. pneumoniae pneumonia, NP necrotizing pneumonia, PE pulmonary embolism, NLR neutrophil-to-lymphocyte ratio, APTT activated partial thromboplastin time, PT prothrombin time

Tab S2: Comparison of inflammatory cytokines among different outcomes

| A SMPP |  |  | |  |
| --- | --- | --- | --- | --- |
| Cytokine | M (Q1, Q3) | Z-value | | P-value |
| IFN-γ 0  1 | 2.44(6.03,24.02)  12.96(6.21,23.78) | -8.15 | | 0.415 |
| IFN-α 0  1 | 1.50(0.96,2.44)  1.51(0.98,2.92) | -1.38 | | 0.167 |
| IL-1β 0  1 | 12.7(5.9,24.61)  11.58(4.62,20.54) | -2.60 | | 0.009 |
| IL-2 0  1 | 1.62(1.15,2.29)  1.78(1.3,2.62) | -4.88 | | <.001 |
| IL-4 0  1 | 1.08(0.65, 1.42)  1.16(0.88, 1.55) | -5.63 | | <.001 |
| IL-5 0  1 | 2.52(1.66, 4.61)  2.27(1.54, 3.88) | -2.66 | | 0.008 |
| IL-6 0  1 | 4.22(2.24, 10.28)  3.65(2.23, 8.75) | -1.05 | | 0.294 |
| IL-8 0  1 | 11.34(1.94, 33.49)  5.35(1.26, 28.45) | -3.5 | | <.001 |
| IL-10 0  1 | 1.89(1.29, 2.99)  2.00(1.29, 3.3) | -2.02 | | 0.044 |
| IL-12p70 0  1 | 1.49(1.12, 1.97)  1.44(1.14, 1,84) | -0.45 | | 0.651 |
| IL-17 0  1 | 3.88(1.73, 11.57)  4.88(2.17, 11.96) | -2.18 | | 0.029 |
| TNF-α 0    1 | 1.29(0.71, 3.36)  1.35(0.76, 2.88) | | -.093 | 0.926 |
| B RMPP |  | |  |  |
| Cytokine | M (Q1, Q3) | | Z-value | P-value |
| IFN-γ 0  1 | 12.18(5.86, 21.09)  13.12(6.44, 24.88) | | -2.44 | 0.015 |
| IFN-α 0  1 | 1.44(0.94, 2.66)  1.52(1.01, 2.88) | | -1.37 | 0.171 |
| IL-1β 0  1 | 11.58(5.27, 19.84)  11.86(4.88, 22.18) | | -0.83 | 0.408 |
| IL-2 0  1 | 1.67(1.18, 2.36)  1.78(1.3, 2.66) | | -3.95 | <.001 |
| IL-4 0  1 | 1.10(0.79, 1.43)  1.16(0.86, 1.55) | | -3.28 | <.001 |
| IL-5 0  1 | 2.27(1.54, 3.8)  2.38(1.58, 4.24) | | -1.46 | 0.145 |
| IL-6 0  1 | 3.65(2.19, 8.83)  3.85(2.25, 9.51) | | -1.47 | 0.140 |
| IL-8 0  1 | 9.77(1.26, 29.6)  5.93(1.33, 29.05) | | -0.72 | 0.474 |
| IL-10 0  1 | 1.83(1.25, 2.95)  2.05(1.32, 3.43) | | -3.57 | <0.001 |
| IL-12p70 0  1 | 1.47(1.13, 1.89)  1.45(1.14, 1.87) | | -0.4 | 0.691 |
| IL-17 0  1 | 4.15(1.89, 11.4)  5.05(2.17, 12.37) | | -2.55 | 0.011 |
| TNF-α 0  1 | 1.28(0.71, 3.19)  1.4(0.78, 2.89) | | -0.72 | 0.472 |

**C PE**

| Cytokine | M | Z-value | P-value |
| --- | --- | --- | --- |
| IFN-γ 0  1 | 12.84(6.1, 23.7)  13.64(6.37, 55.78) | -.453 | 0.650 |
| IFN-α 0  1 | 1.51(0.98, 2.82)  1.34(0.77, 1.69) | -1.527 | 0.127 |
| IL-1β 0  1 | 11.79(5.14, 21.4)  9.49(2.44, 13.16) | -2.105 | 0.035 |
| IL-2 0  1 | 1.74(1.25, 2.52)  1.56(1.15, 2.13) | -.877 | 0.380 |
| IL-4 0  1 | 1.15(0.84, 1.51)  0.96(0.85, 1.2) | -1.367 | 0.172 |
| IL-5 0  1 | 2.34(1.58, 4.05)  1.72(1.46, 2.27) | -2.295 | 0.022 |
| IL-6 0  1 | 3.71(2.23, 9.07)  9.07(3.09, 19.92) | -2.468 | 0.014 |
| IL-8 0  1 | 7.43(1.33, 29.77)  1.56(0.41, 3.12) | -3.039 | 0.002 |
| IL-10 0  1 | 1.97(1.29, 3.21)  1.88(1.25, 3.22) | -.415 | 0.679 |
| IL-12p70 0  1 | 1.46(1.14, 1.87)  1.25(1.05, 1.45) | -2.260 | 0.024 |
| IL-17 0  1 | 4.71(2.1, 11.88)  2.38(1.68, 7.44) | -1.773 | 0.076 |
| TNF-α 0  1 | 1.35(0.74, 3.03)  1.16(0.58, 1.53) | -1.703 | 0.089 |

**D NP**

| Cytokine | M (Q1, Q3) | Z-value | *P*-value |
| --- | --- | --- | --- |
| IFN-γ 0  1 | 12.84(6.1, 23.66)  11.79(8.15, 30.54) | -0.90 | 0.367 |
| IFN-α 0  1 | 1.51(0.98, 2.82)  1.31(0.98, 2.55) | -.068 | 0.499 |
| IL-1β 0  1 | 11.75(5.05, 21.27)  12.45(8.1, 25.28) | -1.07 | 0.283 |
| IL-2 0  1 | 1.73(1.25, 2.52)  1.89(1.44, 2.75) | -1.32 | 0.187 |
| IL-4 0  1 | 1.14(0.83, 1.51)  1.20(0.96, 1.62) | -1.66 | 0.096 |
| IL-5 0  1 | 2.33(1.58, 4.03)  2.41(1.58, 3.83) | -0.31 | 0.760 |
| IL-6 0  1 | 3.71(2.23, 9.07)  6.54(2.88, 18.03) | -2.62 | 0.009 |
| IL-8 0  1 | 7.40(1.33, 29.6)  5.27(1.19, 20.52) | -0.83 | 0.409 |
| IL-10 0  1 | 1.96(1.29, 3.2)  2.34(1.39, 3.66) | -1.31 | 0.190 |
| IL-12p70 0  1 | 1.46(1.13, 1.87)  1.43(1.19, 2.08) | -1.08 | 0.282 |
| IL-17 0  1 | 4.63(2.09, 11.86)  5.37(2.56, 11.57) | -0.45 | 0.656 |
| TNF-α 0  1 | 1.35(0.74, 3.03)  1.33(0.95, 2.71) | -0.59 | 0.557 |

RMPP refractory M. pneumoniae pneumonia, SMPP severe M. pneumoniae pneumonia, NP necrotizing pneumonia, PE pulmonary embolism, IL-2 interleukin-2, IL-4 interleukin-4, IL-5 interleukin-5, IL-6 interleukin-6, IL-8 interleukin-8, IL-10 interleukin-10, IL-17 IL-17interleukin-17, IL-12p70 interleukin-12p70, IL-1β interleukin-1β, IFN-γ Interferon-γ, IFN-α Interferon-α, TNF-α tumor necrosis factor


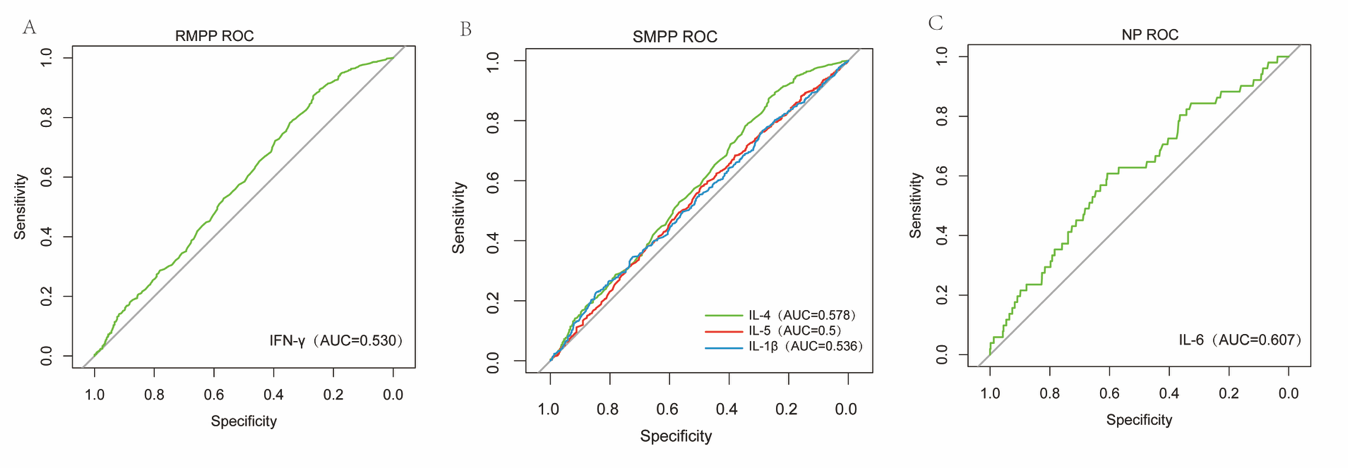


Figure S1: ROC curves were constructed for the evaluation of outcomes using cytokines

RMPP refractory M. pneumoniae pneumonia, SMPP severe M. pneumoniae pneumonia, NP necrotizing pneumonia, IFN-γ (interferon-γ), IL-4 (interleukin-4), IL-5 (interleukin-5), IL-1β (interleukin-1β), IL-6 (interleukin-6)


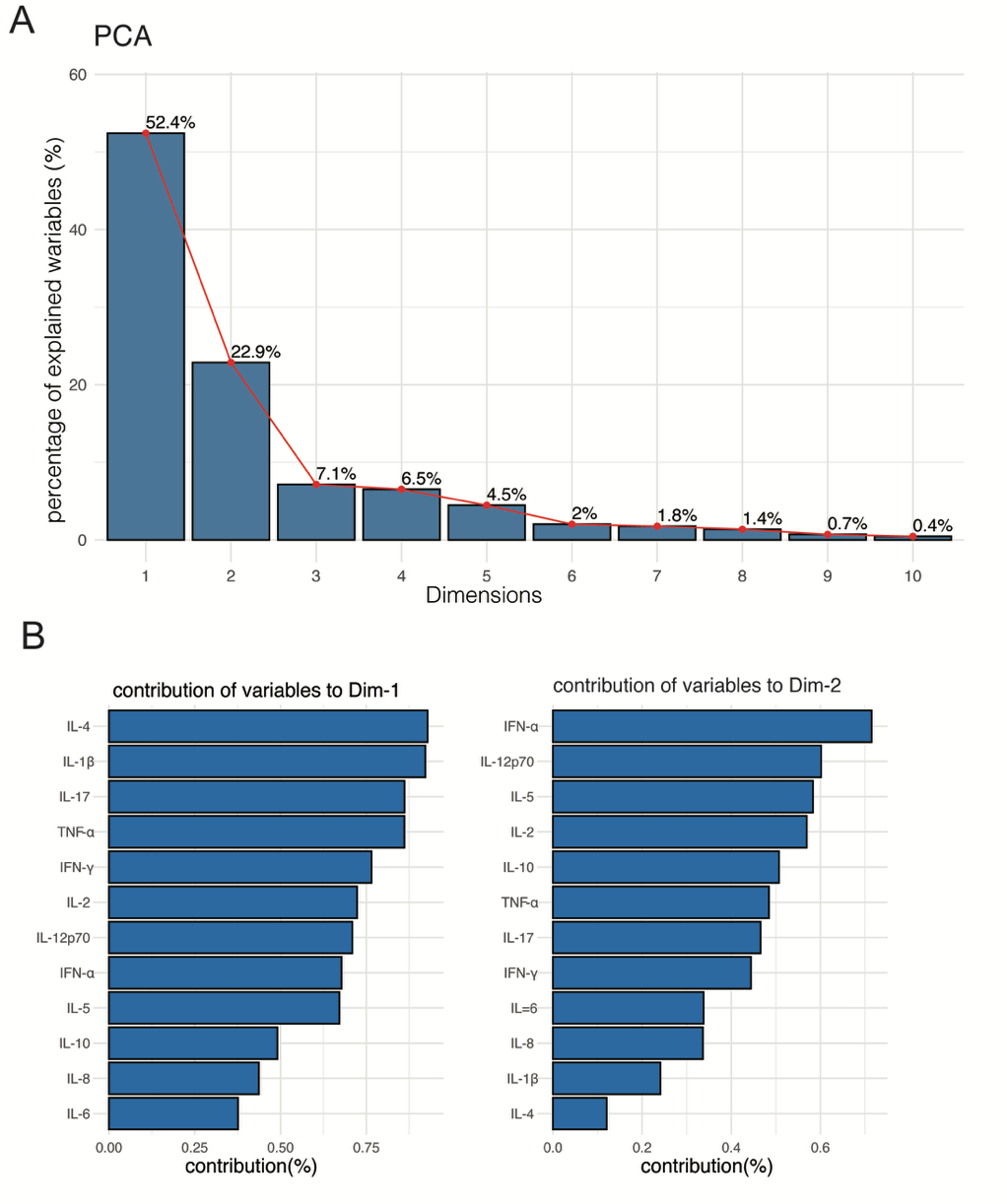


Figure S2: A: PCA identified two components explaining 75.3% (PC1: 52.4%, PC2: 22.9%) variance

B：The cytokine contribution of PCA1 and PCA2

Text S1 Methods: Methodology and quality control for cytokine detection:

Sample collection and processing: 5 mL of peripheral venous blood was collected from each patient within 24 hours of admission. The blood was allowed to coagulate naturally at room temperature for ≥30 minutes, then centrifuged at 1000×g for 10 minutes. Serum was separated immediately for testing; if delayed, serum was aliquoted and stored at -20℃ (avoiding repeated freeze-thaw cycles).

Detection kit and technology: Cytokine levels were quantified using a Multiplex Microsphere Flow Immunofluorescence Luminescence Assay kit (Raisecare Biotechnology, Shandong, China; Cat.). All operations strictly followed the manufacturer’s standard protocol.

The lowest detection limit (LODL) for cytokines was 2.44 pg/mL, and the upper limit of the normal reference value was 10000 pg/mL. The laboratory quality control protocol was as follows: recalibrate when replacing the calibrator lot number or after instrument maintenance/repair. Select high-value samples of the day at irregular intervals, freeze them at -20℃, and use them as inter-day quality controls for testing with the next day's samples. All panels were performed according to the manufacturer’s instructions.
